# Impact of direct oral anticoagulants on blood-brain barrier integrity

**DOI:** 10.1016/j.ibneur.2025.11.007

**Published:** 2025-11-06

**Authors:** Kei Sato, Yoichi Morofuji, Dohgu Shinya, Shinsuke Nakagawa, Takayuki Matsuo

**Affiliations:** a Department of Neurosurgery, Graduate School of Biomedical Sciences, Nagasaki University, 1-7-1 Sakamoto, Nagasaki 852-8501, Japan; b Department of Rehabilitation, Nagasaki University Hospital, 1-7-1 Sakamoto, Nagasaki 852-8501, Japan; c Department of Neurosurgery, SHOWA Medical University Hospital, 1-5-8 Hatanodai, Shina-gawa-ku, Tokyo 142-8666, Japan; d Department of Pharmaceutical Care and Health Sciences, Faculty of Pharmaceutical Sciences, Fukuoka University, 8-19-1 Nanakuma, Jonan-ku, Fukuoka 814-0180, Japan

**Keywords:** Blood brain barrier, Direct oral anticoagulant, Apixaban, Stroke, Hemorrhagic transformation, Transendothelial electrical resistance

## Abstract

**Introduction:**

In cerebral infarction, major prognostic factors include hemorrhagic transformation and cerebral edema, both partially associated with blood-brain barrier (BBB) disruption. Anticoagulation therapy, especially with direct oral anticoagulants (DOACs), is linked to these outcomes, suggesting that the effect of DOACs on BBB integrity may influence stroke prognosis and recovery.

**Methods:**

Two in vitro BBB models—a monolayer model and a co-culture model—were constructed using rat brain endothelial cells (RBECs), pericytes, and astrocytes. Each model was treated with one of three DOACs: apixaban, rivaroxaban, or dabigatran. Transendothelial electrical resistance (TEER) was measured to assess BBB integrity.

**Results:**

In the monolayer model, TEER increased significantly with 10 μM apixaban and decreased significantly with 50 μM dabigatran. In the co-culture model, a significant TEER reduction was observed in the 10 μM dabigatran group, while no significant differences were found for apixaban or rivaroxaban.

**Conclusion:**

These preliminary findings suggest that apixaban may help preserve BBB integrity, while dabigatran at certain concentrations may compromise it.

## Highlights

• Apixaban may preserve blood–brain barrier (BBB) integrity.
• High concentrations of dabigatran may impair BBB integrity.
• Differences in BBB protection among DOACs may contribute to the risk of hemorrhagic infarction.

## 1 Introduction

Damage to the BBB in cerebral infarction has been linked to the onset of hemorrhagic transformation and cerebral edema, both of which are significant predictors of poor prognosis. While we have demonstrated the BBB-protective effects of fasudil (Sato et al., 2022), pitavastatin (Morofuji et al., 2010), and cilostazol (Horai et al., 2013), no drugs with clinically proven BBB-protecting properties have been approved to date. Therefore, there is a pressing need for new therapeutic breakthroughs in this area.

Anticoagulation is a well-established treatment for nonvalvular atrial fibrillation (NVAF), the main cause of cardiogenic cerebral embolism, and is also closely associated with the risk of cerebral hemorrhage and hemorrhagic stroke. Historically, warfarin was the first-line treatment for anticoagulation therapy. Currently, four DOACs are available on the market and are widely used as first-line treatments due to their clinical efficacy and safety profiles. While each DOAC exhibits distinct pharma-cological properties, including variations in efficacy and side effect profiles, direct head-to-head comparisons remain limited, leaving their relative clinical priority unre-solved. This study aims to investigate the role of each DOAC in maintaining BBB integrity using an in vitro BBB model.

## 2 Method

**Fig. 1:**
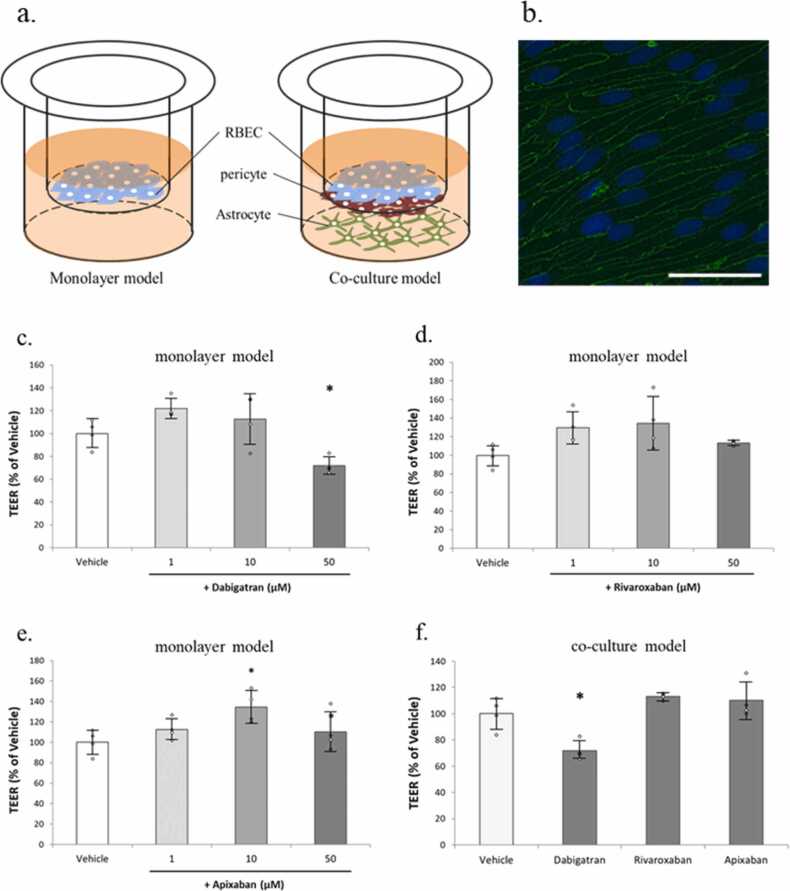
(a) Schematic representation of the in vitro BBB model. In the monolayer model, RBECs are seeded on the upper surface of the transwell insert. In the co-culture model, pericytes are cultured on the underside of the transwell insert, while astrocytes are seeded in the wells of a 24-well plate. (b) Immunofluorescent staining of claudin-5 in RBECs. Scale bar = 50 μm. (c–e) Effect of 24 h DOAC (1, 10, and 50 µM) treatment on the barrier integrity of primary rat brain endothelial cells in the monolayer model. TEER measurement values after treatment. (c) Dabigatran, (d) Rivaroxaban, (e) Apixaban. (f) Effect of 24 h DOAC (10 µM) treatment on the barrier integrity of primary rat brain endothelial cells in the triple co-culture model with primary brain pericytes and astrocytes. TEER measurement values after treatment. n = 4. Independent-samples *t*-test. * p < 0.05.

In this study, we conducted preliminary experiments using an in Vitro BBB model to explore the effects of dabigatran, rivaroxaban, and apixaban on BBB function (Fig. 1a).

Wistar rats were obtained from CLEA Japan. Based on the Guide for the Care and Use of Laboratory Animals from the Ministry of Education, Culture, Sports, Science, and Technology, Japan, all experimental procedures were reviewed by the Institution-al Animal Care and Use Committee of Nagasaki University and finally approved by the University’s president.

RBEC, astrocytes and pericytes were isolated from Wistar rats, as previously de-scribed (Nakagawa et al., 2009). We developed two types of in vitro BBB models: a monolayer model and a co-culture model. For the monolayer model, endothelial cells (2.0 × 10⁵ cells/cm²) were seeded onto the inner surface of collagen-coated inserts (Millicell, 0.4-µm pore size; Millipore) placed in 24-well culture plates after allowing the cells to adhere overnight. Immunofluorescent staining demonstrated that RBECs expressed claudin-5 and grew in a dense, smooth monolayer, indicating an appropriate endothelial phenotype suitable for BBB modeling (Fig. 1b). For the co-culture model, in addition to RBEC pericytes (2.0 × 10⁴ cells/cm²) were seeded on the underside of the inserts, while astrocytes (1.0 × 10⁵ cells/cm²) were cultured on the collagen-coated wells of the 24-well plates. This setup allowed for the establishment of a more physiologically relevant in vitro BBB co-culture system.

Dabigatran, rivaroxaban, and apixaban were purchased from Santa Cruz Biotech-nology (Texas, USA). These compounds were administered onto the luminal (upper) side of the cell culture inserts.

Transendothelial electrical resistance, which reflects the integrity of the BBB mod-el14), was measured using a Millicell® ERS-2 Voltohmmeter (Merck Millipore, USA). The extracellular matrix-coated Millicell inserts were placed in a 24-well plate containing culture medium and were then used to measure the background resistance. The resistance measurements of blank inserts (background resistance) were subtracted from TEER values of inserts with cells. Values are given as Ω × cm2, and data indicate the change in TEER before and after treatment of compounds.

All data are expressed as the means ± standard error of the mean (SEM). Values were compared using analysis of variance followed by *t*-test. A p-value of < 0.05 was con-sidered to be statistically significant.

Kinetics of impedance measurement of the viability of brain endothelial cells after apixaban treatment was monitored by real-time impedance measurement (RTCA-SP, Agilent, Santa Clara, CA, USA). Impedance measurement correlates linearly with cell number, adherence, growth, and viability. The RTCA-SP system (Agilent, Santa Clara, CA, USA) registers the impedance of cells automatically every 10 min. Imped-ance (SI unit: Ω, ohm) is expressed as an arbitrary unit called cell index. For every time point cell index is defined as (Rn − Rb)/15, where Rn is the impedance of the wells containing cells and Rb means the background impedance of the wells contain-ing medium but not cells. hCMEC/D3 brain endothelial cell line were seeded at a cell number of 5 × 103/well onto a 96-well E-plate (Agilent) with golden electrodes at the bottom of the wells, and were kept in the CO2 incubator at 37 °C for 4–5 days. Cells were treated at the beginning of the plateau phase of cell growth with 0, 0.1, 0.3, 1, 3, 10, 30 and 100 μM concentrations of apixaban. Effects of the treatment were followed for 24 h.

## 3 Result

In the monoculture model, groups treated with 1 μM and 10 μM showed a trend toward increased TEER compared to the vehicle group, and a significant increase in TEER was observed only in the 10 μM apixaban group (Fig. 1c-e). In the 50 μM dabigatran group, a significant decrease in TEER was observed (Fig. 1e).

In the co-culture model, treatment was carried out with 10 μM dabigatran, rivaroxa-ban, and apixaban. The dabigatran groups showed a significant decrease in TEER compared to the vehicle group, whereas the group treated with apixaban and rivaroxaban did not exhibit a decrease (Fig. 1f).

**Fig. 2:**
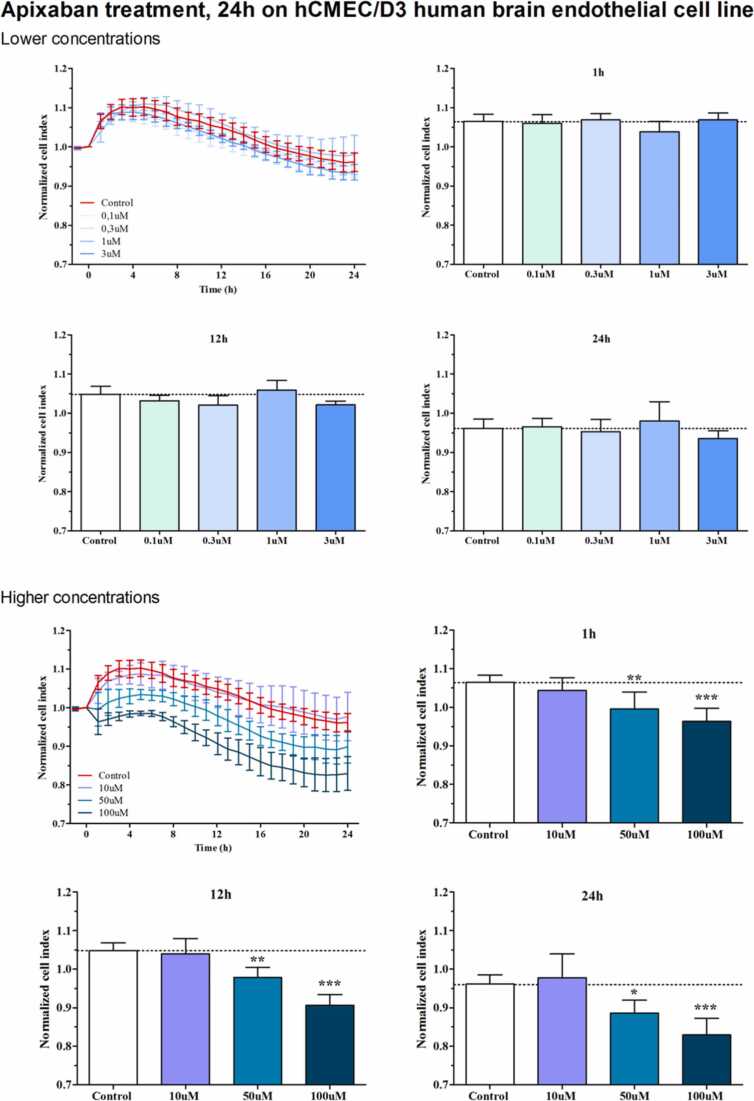
Viability of the hCMEC/D3 brain endothelial cell line after exposure to apixaban at 0, 0.1, 0.3, 1, 3, 10, 30, 50, and 100 μM. Effects were monitored for 24 h. Supraphysiological concentrations (≥50 μM) produced a significant reduction in cell index within the 24 h observation period.

To investigate the direct effect of apixaban on endothelial cells, impedance meas-urements were performed at various concentrations using the hCMEC/D3 brain endothelial cell line. Real-time cell electronic sensing demonstrated that apixaban did not damage human brain endothelial cells at concentrations up to 10 μM, which is 10 times higher than the plasma concentration observed in human subjects. However, at supraphysiological concentrations of 50 μM and 100 μM, the cell index decreased significantly up to the 24-hour measurement time point. The decrease was approximately 10 % with 50 μM treatment and around 15 % with 100 μM treatment (Fig. 2).

## 4 Discussion

Compared to the direct thrombin inhibitor dabigatran, factor Xa inhibitors—especially apixaban—have been shown to exert a protective effect on BBB function. Clinically, apixaban is associated with a lower bleeding risk compared to other anti-coagulants, a finding that aligns with these results. The risk of cerebral edema and hemorrhagic infarction in the acute phase of stroke is thought to arise from multiple factors; however, apixaban may be a preferred choice from the perspective of BBB protection. Furthermore, in clinical scenarios where anticoagulation must be continued despite an elevated risk of intracranial hemorrhage beyond stroke, the choice of anticoagulant becomes particularly important. In patients with mild traumatic brain injury, the incidence of intracranial hemorrhage has been shown to be significantly lower in those receiving DOACs compared with vitamin K antagonists (Karamian et al., 2024, Karamian et al., 2024). However, this reduction is likely to reflect the predominant impact of direct mechanical injury rather than BBB disruption, and may also be influenced by differences in half-life between VKAs and DOACs as well as the availability and efficacy of reversal agents. Thus, the extent to which BBB protection contributes remains to be clarified. In addition, chronic anticoagulation—predominantly with DOACs—did not adversely affect the occlusion rate or long-term outcomes in patients who underwent endovascular treatment for unruptured intracranial aneurysms (Salih et al., 2023). This observation suggests that, despite being anticoagulants, DOACs are unlikely to exert harmful effects on the vascular endothelium even under the high-stress conditions of endovascular procedures. These findings provide a rationale for the continuation of anticoagulation in such surgical settings. Given these trends, DOACs are expected to be increasingly used in high-risk cases, highlighting the importance of selecting the safest regimen. Nevertheless, although DOACs share similar pharmacological properties, they are distinct agents, and ideally each DOAC should be investigated individually in detail. Although none of the cited studies directly compared individual DOACs, further data are warranted. Taken together, these clinical observations may be in line with our experimental findings, suggesting that apixaban may represent a favorable option for BBB protection; however, further confirmatory studies are essential before drawing firm conclusions. Additionally, long-term BBB dysfunction has been identified as a risk factor not only for stroke but also for conditions such as chronic cerebral hypoperfusion (Tomimoto et al., 1996), hypertension (Verhaaren et al., 2013), hyperglycemia (Wardlaw et al., 2003), inflammation (Engelhardt et al., 2016), and aging. Given that DOACs are typically prescribed for prolonged use, their potential long-term impact on the BBB may influence these associated pathologies.

The protective effect on the BBB is mediated by both direct actions on endothelial cells and indirect mechanisms involving interactions with pericytes and astrocytes (Sato et al., 2022). Our preliminary findings indicate that both pathways contribute to this effect. Similar in vitro BBB models, particularly tri-culture systems composed of endothelial cells, pericytes, and astrocytes, have been widely utilized for pharmacological screening and mechanistic studies of barrier regulation. These models are recognized as reliable platforms for evaluating endothelial barrier function under controlled conditions, allowing the investigation of both direct and paracrine-mediated effects on BBB integrity (Carvey et al., 2009, Nakagawa et al., 2007, Keep et al., 2017​). DOACs enhanced TEER at concentrations of 1–10 μM; however, a trend toward decreased TEER was observed at 50 μM, potentially due to cytotoxicity caused by excessive drug concentrations. The maximum plasma concentration of apixaban is reported to be 716 ng/mL, corresponding to 1.56 μM (Frost et al., 2013). Therefore, it is possible that plasma concentrations within the range of 1–10 μM may persist for several hours in vivo study, further investigations are required to determine whether such concentrations have comparable effects on endothelial cells in vivo. In viability experiments with hCMEC/D3, which have properties similar to RBEC, apixaban demonstrated a decrease in TEER at concentrations above 50 μM compared to concentrations up to 10 μM. Activated factor Xa has been identified as an inflammatory mediator, acting on endothelial cells through the activation of protease-activated receptors (PARs) and effector cell protease receptor-1 (EPR-1), and promoting inflammation by generating thrombin from prothrombin in endothelial cells and pericytes (Esmon, 2014, Bono et al., 2000). These results suggest that FXa inhibitors may protect the BBB integrity through multiple pathways, and further explain why FXa inhibitors are more effective at protecting the BBB than dabigatran, which only inhibits thrombin. However, the precise mechanisms underlying these effects remain unclear, and the distinctions between apixaban and rivaroxaban have yet to be fully elucidated. Furthermore, this study has limitations, including a small sample size and the absence of evaluation of edoxaban, highlighting the need for further investigation.

In conclusion, our findings suggest that apixaban may exert a more favorable BBB-protective effect compared to dabigatran and rivaroxaban, potentially reducing the risk of cerebral edema and hemorrhagic infarction in the acute phase of stroke, as well as mitigating the long-term consequences of BBB dysfunction.

## Compliance with ethical standards

All animal procedures were conducted in accordance with the Guide for the Care and Use of Laboratory Animals published by the Ministry of Education, Culture, Sports, Science, and Technology, Japan. All experimental protocols were reviewed and approved by the Institutional Animal Care and Use Committee of Nagasaki University and were finally approved by the university president.

Approval number: 1807131462–2

## Grant support

None.

## CRediT authorship contribution statement

**Yoichi Morofuji:** Writing – review & editing, Supervision, Methodology, Conceptualization. **Takayuki Matsuo:** Supervision. **Kei Sato:** Writing – review & editing, Writing – original draft, Visualization, Software, Resources, Project administration, Methodology, Investigation, Formal analysis, Data curation. **Dohgu Shinya:** Validation, Supervision, Methodology, Conceptualization. **Shinsuke Nakagawa:** Validation, Supervision, Methodology, Investigation.

## Declaration of Competing Interest

The authors declare that they have no known competing financial interests or personal relationships that could have appeared to influence the work reported in this paper.
